# Clonal Selection and Evolution of HTLV-1-Infected Cells Driven by Genetic and Epigenetic Alteration

**DOI:** 10.3390/v14030587

**Published:** 2022-03-12

**Authors:** Makoto Yamagishi, Yutaka Suzuki, Toshiki Watanabe, Kaoru Uchimaru

**Affiliations:** 1 Laboratories of Tumor Cell Biology, Department of Computational Biology and Medical Sciences, Graduate School of Frontier Sciences, The University of Tokyo, Tokyo 108-8639, Japan; uchimaru@edu.k.u-tokyo.ac.jp; 2 Laboratories of Systems Genomics, Department of Computational Biology and Medical Sciences, Graduate School of Frontier Sciences, The University of Tokyo, Chiba 277-8561, Japan; ysuzuki@edu.k.u-tokyo.ac.jp; 3 Department of Practical Management of Medical Information, Graduate School of Medicine, St. Marianna University, Kawasaki 216-8511, Japan; tnabe@marianna-u.ac.jp

**Keywords:** HTLV-1, genome, epigenome

## Abstract

T cells infected with human T-cell leukemia virus type 1 (HTLV-1) acquire various abnormalities during a long latent period and transform into highly malignant adult T-cell leukemia-lymphoma (ATL) cells. This can be described as “clonal evolution”, in which a single clone evolves into ATL cells after overcoming various selective pressures in the body of the infected individuals. Many studies have shown that the genome and epigenome contain a variety of abnormalities, which are reflected in gene expression patterns and define the characteristics of the disease. The latest research findings suggest that epigenomic disorders are thought to begin forming early in infection and evolve into ATL through further changes and accentuation as they progress. Genomic abnormalities profoundly affect clonal dominance and tumor cell characteristics in later events. ATL harbors both genomic and epigenomic abnormalities, and an accurate understanding of these can be expected to provide therapeutic opportunities.

## 1. Introduction

Genetic abnormality is the main feature of cancer and greatly alters cell fate. The proliferative cycle is essential in the early stages of cancer development since DNA replication errors and abnormal repair functions associated with cell division are the main requirements for development. Through the analogical process of “natural selection” advocated by Charles R. Darwin [1], cells that acquire genetic abnormalities become dominant under selective pressure as a more adapted population and eventually progress to a pathological state [2,3].

On the other hand, epigenetic abnormalities result from a series of processes regulated by multiple complexes and often do not require excessive cell division as a condition for development [4]. Almost all cancers have been shown to have characteristic epigenetic alterations such that a complex tumorigenic mechanism with “genomic change as a driver” and “epigenetic dysregulation as an essential background” have been postulated [5,6]. These processes can be characterized as a blueprint and its use. The critical point is that both processes are heritable characteristics and can be passed on to the next generation at the cellular level, which is the essence of forming a population of cells with the same characteristics.

Human T-cell leukemia virus type 1 (HTLV-1) mainly infects CD4+ T cells and disrupts host signaling pathways and gene expression patterns with viral gene products such as Tax and HBZ [7,8]. As a result, many immortalized infected clones are established in the early stage of infection. During the subsequent long latent period, infected cells that have accumulated abnormalities in the host genome and epigenome selectively proliferate, and 3–5% of infected individuals develop adult T-cell leukemia-lymphoma (ATL), and probably less than 1% develop inflammatory diseases such as HTLV-1-associated myelopathy (HAM), HTLV-1 uveitis, infective dermatitis and chronic pulmonary disease [9,10].

ATL is a neoplastic disease with a very poor prognosis in which one of the numerous infected cells evolves clonally over 30 to 50 years. Recent comprehensive genome, epigenome, and gene expression analyses have provided macroscopic views of aggressive type ATL [11]. However, the process of clonal evolution leading to pathogenesis is complex. Therefore, a better understanding of the pathogenic mechanism may be achieved by tracing back to the cells infected with HTLV-1 and further increasing the resolution of the analysis to the cellular level.

A series of intensive studies shows that HTLV-1 causes the immortalization of infected cells by viral gene products after infection, resulting in abnormal proliferation. Individual studies of oncogenic viruses are essential in pathophysiology and drug discovery. Furthermore, the study of viral tumorigenesis is an excellent model for understanding the nature of cancer initiation, diversity, clonal selection, and evolution. These viral infections can be regarded as the first hit of a multistep carcinogenic process. Tracing the precancerous cell population formed by the infection along the timeline may help unravel the multistep process.

In this review article, we pick up some of the latest studies on the mechanism of HTLV-1 induced tumorigenesis from the viewpoint of host epigenomic abnormalities, especially modifications that significantly influence gene expression patterns, and clonal evolution with genomic abnormalities, and discuss the future aspects.

## 2. Early Formation of Epigenomic Abnormalities in Infected Cell Populations

The essential nature of epigenetics has been detailed in excellent reviews [4,5,6,12]. The epigenome of a somatic cell can be flexibly altered by various external factors such as environment, aging, or internal factors coming from the genetic level.

One of the most remarkable properties of the epigenome in contrast to the genome is its flexibility. This means that it is susceptible to change, and conversely, it is theoretically possible to restore the epigenome to its original state [13]. This point is of the highest importance from the standpoint of biology and medical science. The establishment of iPS cells that have been achieved by reprogramming somatic cells is a clear example of this theory [14].

**Figure 1:**
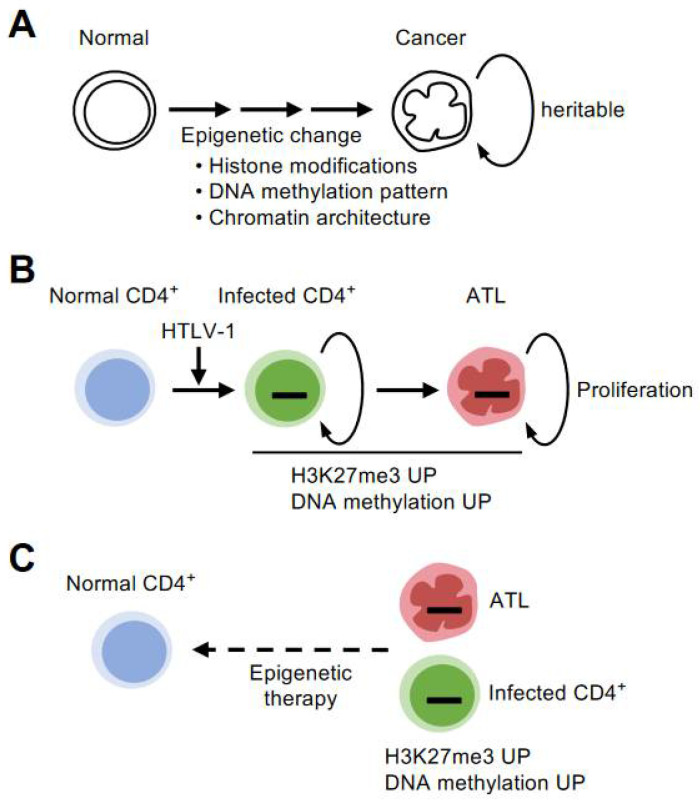
Early formation of epigenetic abnormalities. (**A**) Various abnormalities in epigenetic modifications accumulate during the transformation process from normal cells to tumor cells. The epigenetic changes, such as DNA methylation and histone modifications, can be inherited by the next generation of cells to form populations with the same characteristics. This has a high affinity with the endlessly proliferating nature of cancer. (**B**) T cells infected with HTLV-1 gradually develop into high-grade ATL cells with abnormal accumulation of H3K27me3 and DNA methylation. Such epigenomic abnormalities are common characteristics of infected and highly proliferating ATL cells. (**C**) Epigenetic changes are highly plastic. It is essential to clarify the direction and mechanism of epigenetic abnormalities in infected and tumor cells. Then, by precisely targeting them as therapeutic candidates, the concept of restoring the undesired epigenomic characteristics to their original state can be established.

Another property of the epigenome is its heritability. This is somewhat surprisingly similar to the nature of DNA. Although the inheritance of the epigenome across generations of individuals is controversial, the inheritance of the epigenome from cell to cell is evident; epigenetic changes, such as DNA methylation and histone modifications, once written or erased, can be passed on to the next generation of cells to form populations with the same characteristics [12,13]. This seems to have a close affinity with the endlessly proliferating nature of cancer (Figure 1A).

Several studies have suggested the existence of HTLV-1-triggered epigenetic abnormalities. Their findings show how (1) transcriptome analysis of infected cells purified with specific surface markers and monoclonal tumor cells showed a common expression pattern across cases [15]; (2) miR-31 expression is commonly silenced in all ATL cases, but most cases are not accompanied by gene mutations in the coding region or copy number reduction [16]. miR-31 is also silenced in polyclonal infected cell populations in infected individuals; (3) expression of genes involved in histone modification and chromatin regulation differs significantly between normal and infected cells [17]; and (4) Tax encoded by HTLV-1 interacts with multiple host epigenomic factors [17,18,19,20].

A genome-wide ChIP assay revealed epigenetic changes in infected and ATL cells and demonstrated large-scale changes in trimethylation of the 27th lysine residue of histone H3 molecules (H3K27me3) [17]. In ATL cells, H3K27me3 accumulates and represses the expression of many genes, including tumor suppressor genes, transcriptional regulatory genes, epigenetic-related genes, and microRNA loci. This epigenetic downregulation occurs most dramatically in the acute form of ATL. However, infected cells in smoldering and chronic ATL and HTLV-1 carriers also show gene silencing by H3K27me3 at many loci [17].

An important finding needs to be pointed out here. The enzyme that catalyzes H3K27me3, an enhancer of zeste homolog 2 (EZH2), physically interacts with HTLV-1 Tax. This interaction is thought to disrupt the scope of target genes originally regulated by EZH2, resulting in a genome-wide change in the pattern of H3K27me3. By inactivating the function of EZH2 with an inhibitor during the process of immortalization by Tax, it is possible to stop the growth of infected cells by Tax [17]. This is an example that links the importance of changes in H3K27me3 at the molecular level to the process of infected cell proliferation (Figure 1B).

In addition to EZH2, the interaction of Tax with other histone-modifying enzymes such as SUV39H1 and HDAC1 has been reported [18,19]. Similarly, an interaction between HBZ protein, which is encoded by HTLV-1 antisense strand, and SWI/SNF chromatin remodeling family has also been reported [21,22]. These interactions have been shown to have a role in regulating the provirus. However, it is not difficult to speculate that the critical significance of epigenomic changes in regulating the host’s vast genome is also crucial in controlling the fate of infected cells.

In addition, the insertion of the HTLV-1 genome has been implicated as a possible effect on the host epigenome. CTCF-binding sequences on the provirus suggest a new relationship between the provirus and host epigenome [23]. Some studies have shown that HTLV-1 disrupts the host chromatin structure by forming a loop between the provirus and the host genome, and the loop depends on the critical chromatin architectural protein CTCF [23,24,25,26]. Although it remains unclear how these changes contribute to the characteristics of infected cells, clonal structure, and pathogenesis of ATL, the impact of such insertional mutagenesis on the host epigenome is an essential insight into the pathogenicity of HTLV-1.

## 3. Targeting Epigenomic Abnormalities to Combat Infected Cells

A polycomb family is a group of factors that regulate chromatin structure using H3K27 methylation. Polycomb repressive complex 2 (PRC2), which contains either H3K27 methyltransferases EZH1 or EZH2, serves as the basic unit for inducing H3K27me3 [27]. The binding patterns of EZH1 and EZH2 on all chromatin in ATL cells indicate that both enzymes cooperatively cause the accumulation of H3K27me3 [28].

Interestingly, when referring to the expression patterns of various cell types, the expression of EZH1 and EZH2 shows an inverse correlation. For example, undifferentiated hematopoietic stem cells have high H3K27me3 that is induced by high EZH2 expression to maintain their multipotency. On the other hand, expression of EZH1 is high, and that of EZH2 is low in mature lymphocytes. This indicates that either EZH1 or EZH2 functions primarily in the formation and maintenance of the necessary H3K27me3 pattern. When EZH2 is overexpressed in mature T cells with high EZH1 expression, the coexistence and function of EZH1-PRC2 and EZH2-PRC2 are phenomenally consistent with the overall increase in H3K27me3 in tumor cells [28]. This finding theoretically indicates that inhibition of EZH2 alone is insufficient. Compared to conventional single inhibitors of EZH2, a new class of inhibitors that can simultaneously inhibit EZH1 and EZH2 has been shown in model cell lines and in vivo models to more efficiently elicit anti-ATL cell effects by eliminating accumulated H3K27me3 and ably restoring target gene expression [28].

Notably, preclinical studies have shown the concept that targeting both EZH1 and EZH2 can normalize the accumulation of H3K27me3 not only in high-grade tumor cells but also in cells in a precancerous state infected with HTLV-1 present in the peripheral blood of infected individuals [28] (Figure 1C). Recently, abnormalities in methylated DNA, which similarly suppresses gene expression, have been comprehensively investigated [11,29]. Similar to the H3K27me3 abnormality, DNA methylation has also been shown to be abnormal in infected cells of carriers that have not yet developed the disease. New therapeutic strategies targeting such early stage epigenomic abnormalities are expected to be one of the major challenges for early therapeutic intervention for diseases with poor prognosis.

## 4. Overview of Genetic Characteristics in ATL

How does a characteristic infected cell population, formed by viral genes and a series of epigenetic changes, subsequently evolve into ATL? This is revealed by the comprehensive genetic analyses of the ultimately evolved ATL cells.

Genomic studies of ATL have a long history, and early results of chromosome analysis showed chromosomal abnormalities in 96% of cases [30]. Subsequent comprehensive analysis using the comparative genomic hybridization (CGH) method has revealed the high frequency of genomic abnormalities in acute and lymphoma types of ATL and their correlation with prognosis [31].

A comprehensive study by Kataoka et al. has provided an overview of genetic mutations and copy number variations (CNV) in ATL cells [11]. The most significant characteristic is a high integration of genetic abnormalities in the T-cell receptor (TCR)/NF-κB signaling pathway. More than 90% of the cases had at least one genetic abnormality in this pathway, with a large number of gain-of-function mutations, including *PLCG1* (36%), *PRKCB* (33%), *CARD11* (24%), *VAV1* (18%), *IRF4* (14%), and *FYN* (4%) mutations. Other mutations include signaling factors such as *STAT3* (21%) and *NOTCH1* (15%), transcription factors such as *IKZF2* (35%), *TP53* (18%), *GATA3* (15%), and *IRF4* (14%), epigenetic factors such as *TET2* (8%) and *EP300* (6%), chemokine receptors such as *CCR4* (29%) and *CCR7* (11%), and structural variants in the *CD274* (encoding PD-L1) (27%), which is important for immune evasion, were also identified [32].

**Figure 2:**
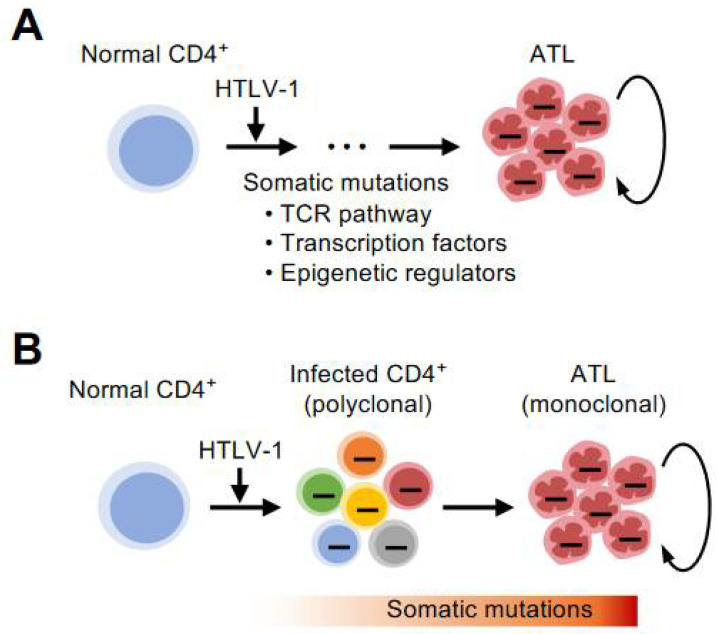
Genetic characteristics in ATL. (**A**) A number of genomic abnormalities are detected in ATL cells. These traces imprinted on the DNA indicate abnormalities necessary for evolution into highly malignant clones. (**B**) Some critical genetic mutations are detected in diverse populations of infected cells before disease onset by deep sequencing. Specific clones are selected to evolve into ATL cells.

Although somewhat different in frequency and pattern, Shah et al. reported similar data [33]. The frequency of abnormalities in epigenetic-related genes is higher in North American cases. Thus, the genomic abnormalities of ATL are diverse, and the mode of clonal growth of infected cells is expected to be extremely complex in each case (Figure 2A). More recently, large-scale whole-genome sequencing (WGS) has provided a complete picture of the genomic features of ATL, including not only single nucleotide variants and short Indels but also mutations in noncoding regions and structural abnormalities [34]. These new views were statistically revealed by large-scale analysis that genomic aberrations are essential in the process of evolution to the eventual monoclonal ATL.

## 5. Estimating the Clonal Evolution of Infected Cell Populations

The most obvious feature of the genome of ATL is that it is characterized by many abnormalities in the TCR pathway. However, the genomic abnormalities in each case are highly diverse, and it is at the same time clear that there is no single mechanism for the development of ATL. After HTLV-1 infection, a polyclonal population of infected cells is formed by viral gene products and epigenomic disorders. Over the next several decades, a single infected clone is thought to gain dominance and proliferate through various genomic aberrations. So then, what mechanisms are involved in clonal selection during this long latent period? A possible approach is to consider genomic mutations as patterns and address them quantitatively by setting up axes such as time scale, mutant clone size, and disease history.

One important implication is that many of the genetic abnormalities can be detected before the disease onset. Rowan et al. used deep sequencing to go back in time and detect most of the genetic abnormalities during the carrier phase and showed that they clonally expanded as the disease progressed [35]. Marçais et al. reported the evolution of ATL cells with various genetic mutations in progression from indolent type to aggressive type and before and after chemotherapy [36]. Such early detection of genetic abnormalities and traces of clonal evolution have been confirmed in our Japanese cohort [37]. These observations are consistent with the theory of natural selection of species, and lead to the conclusion that cancer evolves through clonal selection and propagation (Figure 2B).

This disease has three features that provide analytical advantages in tracing and understanding this evolutionary process. The first is that the “first hit” in clonal evolution is defined as “HTLV-1 infection”. In general, cancer cells of origin are formed by various internal factors (hereditary tumors, SNPs, etc.) and external factors (diet, alcohol, smoking, stress, etc.). However, it is not easy to detect them early in clinical specimens and study their characteristics and mechanisms of cancer development. On the other hand, ATL always has a background of direct effects of HTLV-1 infection, and its interrelationship with subsequent clonal evolution can be studied more deeply.

Second, by analyzing the viral genome inserted into the host genome (provirus), it is possible to distinguish individual polyclonally infected cells. It has been shown that each infected cell clone has a randomly inserted virus somewhere in the host genome of approximately 6 billion base pairs, and by using this insertion site information as an ID, each clone can be distinguished and traced with extreme accuracy. Furthermore, the size of each clone can be easily estimated by quantifying the chimeric reads between the end of the provirus and the host genome, or the paired-end reads spanning the two, and other analysis techniques have also been established [38,39,40]. In addition, data on internal sequences, deletions, and mutations in the viral genome and proviral ends can be obtained to analyze the phylogenetic tree of the virus and its relationship to host immunity [41].

Third, a highly accurate analytical method using specific surface antigens of HTLV-1-infected cells has been established; surface antigens such as CD4+, CD25+, CCR4+ [42], and CADM1+ [43], have been identified, and the expression of CD7 [44] and CD26 [45] decreases with progression to ATL. The authors developed a flow cytometric method (HAS-Flow method) [15], which enables objective evaluation and isolation of infected cells without a morphological diagnosis, noting that infected cells are enriched in the CD4+/CADM1+ population in peripheral blood of infected patients and that CD7 expression decreases significantly with progression to ATL. By using this method, not only monoclonal ATL cases with proliferating tumor cells but also smoldering/chronic ATL before acute transformation and infected cells in pre-symptomatic HTLV-1 infected carriers can be sensitively detected and fractionated.

## 6. Diversity of Infected Cells and Clonal Competition

Diversity is the essence of cancer. In the case of solid tumors, methods to estimate diversity and evolution are often used, mainly by sequencing analysis of a large number of pathological regions. On the other hand, in the case of circulating hematological tumors, it is difficult to read out the exact clonal composition based only on genomic information from a single time point in peripheral blood. We recently analyzed a time series of clinical specimens from the same infected individuals over a period of about ten years. We succeeded in depicting the process of competition among individual infected clones, followed by the acquisition of genomic abnormalities by some infected clones and the increase in clone size [37]. High-depth genomic and clonality analyses revealed that the peripheral blood of infected individuals contains an extremely heterogeneous population consisting of numerous different infected cell clones distinguished by proviral integration sites, as well as subclones with different genetic mutation patterns.

**Figure 3:**
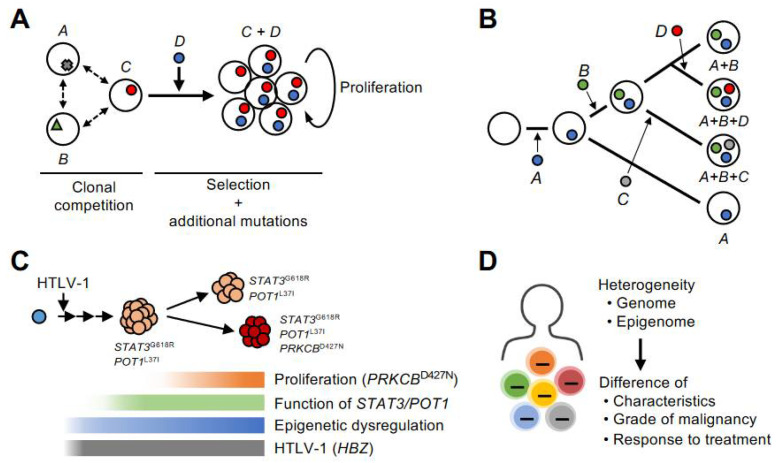
Clonal evolution mechanism by genome and epigenome. (**A**) In a heterogeneous population consisting of cells with various abnormalities, the clone with the greater dominance is selected through a clonal competition. (**B**) Infected clones evolve and adapt to their environment by acquiring genetic mutations in a stepwise manner during clonal selection. ATL is a monoclonal malignancy in which a single infected cell has evolved through a multistep process. However, the tumor cell population is not homogeneous at the genome, epigenome, and other properties, but is composed of diverse subclones. (**C**) This schematic model provides an example of the evolutionary process to acute-type ATL. In this case, the emergence of a high-grade clone that acquired the *PRKCB* mutation led to the development of acute type disease. The effect of genetic abnormality is expressed as a gene expression pattern in each subclone. The accumulation of H3K27me3 acquired before the subclonal formation and the associated expression abnormalities are detected as common characteristics in the subclones. (**D**) Genomic and epigenomic heterogeneity is reflected in differences in the characteristics of infected and tumor cells, their grade of malignancy, and their responsiveness to therapy.

Accurately capturing the properties of each clone from a heterogeneous population is difficult using conventional bulk methods. Single-cell RNA-seq (scRNA-seq) is a methodology that can overcome these technical challenges. Rather than estimating clonal structure by extrapolating data from bulk samples, it physically distinguishes cells and sequences each cell individually. We have constructed a new pipeline to identify infected cell populations by extracting viral genes (HBZs) that are constantly expressed in infected cells from sequencing reads and mapping them to clustering. In addition, an analysis method that can identify clones with genetic mutations by extracting mutant RNA reads was also incorporated into the pipeline. Using this method, it is possible to distinguish each mutated subclone by clustering each genetic mutation [37]. A new insight detected by using this platform was a competition between different ATL subclones (Figure 3A).

The tumor cells in the peripheral blood of ATL patients were not homogeneous but were a heterogeneous population consisting of multiple infected clones with different characteristics. The characteristics (i.e., gene expression patterns) of each infected clone were consistent with the patterns of genetic characteristics, suggesting that genomic abnormalities define the fundamental clonal structure. Analysis of these cases at different times showed that the clone prevalence ratio was altered, with one clone increasing relative to the other. This can be described as a process of competition and selection between multiple infected clones.

Gene expression patterns suggested that the clones that had actually increased relative abundance were more proliferative than those that were initially dominant. The use of different signaling pathways was also revealed. These data represent a moment of clonal selection in the infected cell population and the fact that the characteristics of each clone correlate well with the pattern of genetic abnormalities.

## 7. Genetic Abnormalities in Multistep Tumorigenesis

Another important finding is the gradual acquisition of genetic abnormalities. A multistep evolutionary process with genetic abnormalities was detected by following the same patient along the time axis [35,36,37] (Figure 3B). Integrative analysis with scRNA-seq method revealed that the new genetic mutations led to the progression of very aggressive features compared to the existing clone [37]. It should also be noted that in this case, there was a rapid increase in abnormal lymphocytes that corresponded with this clonal evolution. These observations warrant the importance of genetic mutations in the evolution of ATL cells. The integration of genomic and transcriptomic data also reveals that genetic mutations significantly impact the properties of ATL cells.

The critical insight here is that the clones before acute transformation have already acquired some genetic abnormalities and have increased clone size compared to other infected cell populations. This premalignant clone showed a distinctly abnormal gene expression pattern compared to normal cells. This indicates that, although this infected clone has become dominant in the population, it has not progressed to the final stage and appears to be in the process of natural selection. This situation is probably common in carriers with increasing numbers of infected cells and patients with indolent types of ATL. The difference between clones that progress to the final stage and those that stop before the final stage cannot be accurately predicted at present. If we can identify the characteristics of malignant clones based on patterns of gene abnormalities, gene expression, and epigenomic abnormalities, it will be beneficial for prognosis prediction and therapeutic drug development.

## 8. Clonal Evolution Mechanism by Genome and Epigenome

How are polyclonal cell populations evolving using genomic and epigenomic abnormalities? An important suggestion is that the epigenome is a common feature across cases. In the single-cell analysis, epigenomic abnormalities were commonly detected in comparative analysis of competing cell populations and cells before and after progression [37]. Gene suppression by H3K27me3 is detected in monoclonal cells derived from ATL patients, polyclonal cells derived from HTLV-1 carriers, and Tax-expressing cells [17,28]. In addition to repressive epigenomes, many genes are overexpressed independent of the pattern of genetic abnormalities in the infected polyclonal cell population, including *CCR4* and *CADM1*. It has also been reported that some of these are induced by HBZ [7,8,46] and superenhancer formation [47]. The evidence suggests that HTLV-1 is involved in the initial formation and maintenance of the aberrant epigenome. Targeting the common epigenomic dysregulation would provide broad and durable therapeutic benefits. In addition, suppressing the early polyclonal population might reduce the opportunities for subsequent evolution into more malignant clones.

The genomic abnormalities detected in ATL cases are very complex. Although they share common features such as the TCR signaling pathway, they are more complex than other typical leukemias and some solid tumors and appear to involve clonal individuality rather than common properties. It is not clear what combination of genetic abnormalities is responsible for the underlying hallmarks of tumor cells, such as abnormal proliferation and evasion from the apoptotic form of regulated cell death (RCD). The fact that the pattern of genetic abnormalities varies among cases may indicate redundancy among genetic abnormalities. Considering the direct effects of viral genes and the commonality among clones, epigenomic disorders are thought to begin forming early after infection and evolve into ATL through further changes and accentuation as they progress (Figure 3C). To the extent that they can be detected, genomic abnormalities profoundly affect clonal selection at the time of clonal dominance in events later than epigenomic abnormalities. In addition, chromosomal instability and the resulting CNVs, coupled with point mutations, can critically affect clonal dominance. Tumor cell populations formed by multiple aberrations in the genome and epigenome are not homogenous and produce differences in characteristics, malignancy, and responsiveness to therapy (Figure 3D). This idea is consistent with the clonal evolution of other cancers [2,3]. Diversity within the infected cells will be an important topic of future studies.

It should be noted that the genomic variations referred to here are only within the range that can be considered with the current sensitivity of the analysis. It cannot be determined from which point of time they are introduced. Genomic instability caused by HTLV-1 has been pointed out, and there may be genomic variations that do not lead to clonal expansion beyond a distinguishable level. Many passenger mutations and subclonal structures caused by neutral evolution have been detected in ATL cases. The function of mutations in noncoding regions is also largely unknown.

It is also necessary to consider the role of selective pressure. Environmental factors such as oxidative stress, nutrient conditions, and therapeutic agents may contribute to the evolution of polyclonal populations resulting from HTLV-1 infection. In addition, evidence of immunological selective pressure is certainly documented at the genomic level, as exemplified by structural variation in *PD-L1* [32]. Because Tax is highly immunogenic, cells expressing Tax are selectively eliminated by CTLs. CTL is probably one of the most influential host determinants of host immunity that regulates infected cells [48,49].

The evolutionary path of T cells infected with HTLV-1 should have been inscribed in the ATL cells that appear as the disease. Characterizing the heterogeneous ATL cell population may help us identify opportunities for durable therapeutic intervention. A multifaceted understanding of the path from infection to ATL and HAM from the elements of virology, genomics, epigenomics, and host factors would lead to a significant advance in managing HTLV-1 infectious diseases.

## Data Availability

Not applicable.
